# Marked Differences in Human Melanoma Antigen-Specific T Cell Responsiveness after Vaccination Using a Functional Microarray

**DOI:** 10.1371/journal.pmed.0020265

**Published:** 2005-09-20

**Authors:** Daniel S Chen, Yoav Soen, Tor B Stuge, Peter P Lee, Jeffrey S Weber, Patrick O Brown, Mark M Davis

**Affiliations:** 1Department of Internal Medicine/Division of Oncology, Stanford University, Stanford, California, United States of America,; **2**Howard Hughes Medical Institute, Stanford University, Stanford, California, United States of America,; **3**Department of Biochemistry, Stanford University, Stanford, California, United States of America,; **4**Department of Medicine, Stanford University, Stanford, California, United States of America,; **5**Norris Cancer Center, University of Southern California, Los Angeles, California, United States of America,; **6** Department of Microbiology and Immunology, Stanford University, Stanford, California, United States of America; University of EdinburghUnited Kingdom

## Abstract

**Background:**

In contrast to many animal model studies, immunotherapeutic trials in humans suffering from cancer invariably result in a broad range of outcomes, from long-lasting remissions to no discernable effect.

**Methods and Findings:**

In order to study the T cell responses in patients undergoing a melanoma-associated peptide vaccine trial, we have developed a high-throughput method using arrays of peptide-major histocompatibility complexes (pMHC) together with antibodies against secreted factors. T cells were specifically immobilized and activated by binding to particular pMHCs. The antibodies, spotted together with the pMHC, specifically capture cytokines secreted by the T cells. This technique allows rapid, simultaneous isolation and multiparametric functional characterization of antigen-specific T cells present in clinical samples. Analysis of CD8+ lymphocytes from ten melanoma patients after peptide vaccination revealed a diverse set of patient- and antigen-specific profiles of cytokine secretion, indicating surprising differences in their responsiveness. Four out of four patients who showed moderate or greater secretion of both interferon-γ (IFNγ) and tumor necrosis factor-α (TNFα) in response to a gp100 antigen remained free of melanoma recurrence, whereas only two of six patients who showed discordant secretion of IFNγ and TNFα did so.

**Conclusion:**

Such multiparametric analysis of T cell antigen specificity and function provides a valuable tool with which to dissect the molecular underpinnings of immune responsiveness and how this information correlates with clinical outcome.

## Abstract 2

Patients vaccinated with melanoma-associated peptides show a wide diversity of responses, analysis of which may help us understand the differing clinical responses to such vaccines.

## Introduction

Antigen-specific cellular immune responses are mediated by αβT cell receptor (TCR)-bearing T cells that recognize specific peptides bound to major histocompatibility complex (MHC) molecules on the surfaces of other cells. These T cells form a major part of the adaptive immune response. CD8+ T cells mediate direct lysis of infected or aberrant cells, whereas CD4+ T helper cells modulate antibody (B cell) responses and those of other cells. T cells may become activated following antigen recognition and respond by secreting soluble factors, which include mediators of target cell lysis, pleiotropic effector factors, growth factors, and inflammatory and regulatory cytokines (Table 1). This is a highly regulated and complex process. In many cases, antigen recognition by primed CD8+ T cells leads to the lysis of cellular targets and the release of inflammatory cytokines. Alternatively, this response may be partially or completely anergic.

**Table 1:**
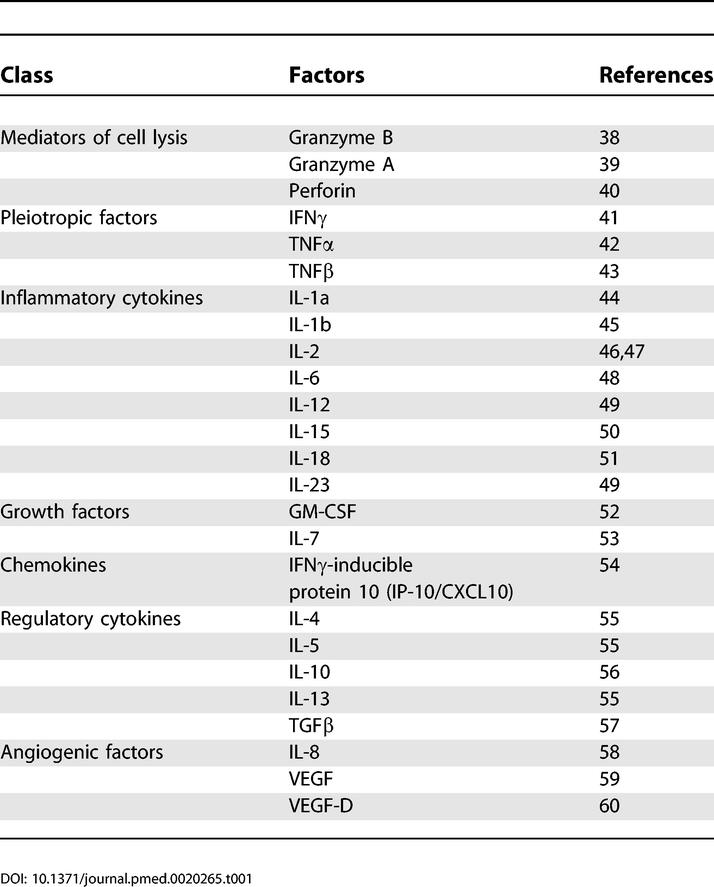
Factors Secreted by Lymphocytes or Other Cells of the Immune System

For many years, investigators have sought to direct T cell responses against tumors by vaccination [1]. These efforts have been greatly aided by the discovery of many peptide antigens that are displayed on MHC molecules on the surface of tumor cells and that have been shown to elicit T cell responses both in vitro and in vivo [2,3]. This discovery has given rise to a variety of strategies, including protein and peptide vaccination [4], adoptive cellular therapy [5], cytokine therapy (i.e., interleukin [IL]-2, granulocyte-macrophage colony-stimulating factor [GM-CSF], interferon [IFN] α) [6–8], and immune response modifiers such as anti-CTLA4 [9,10]. Despite intense efforts, the success of most of these protocols has been mixed. Although in many cases, specific T cell responses can be generated in patients (or expanded ex vivo and reintroduced intravenously), they are not usually effective against the tumor. A large part of the problem may be that most of these tumor-associated antigens are normal “self” peptides, and responses may be naturally suppressed. In this context, it is important to monitor the precise functional status of T cells that are elicited by a particular immunization protocol, and to determine what conditions result in T cells that are the most effective in bringing about clinically significant results. For this purpose, the ability to track antigen-specific T cells with peptide-MHC (pMHC) tetramers [11] has been an important tool in the identification and characterization of lymphocytes capable of recognizing specific tumor antigens. This technique, together with other assays (e.g., intracellular cytokine staining, CD107, ELISpot, killing assay) have been used to try to address T cell function [12–15]. However, these assays are labor intensive, require large quantities of clinical peripheral blood mononuclear cell (PBMC) specimens for a comprehensive analysis, have poor spatial resolution and/or low sensitivity for secreted responses, and do not address the growing need to track multiple T cell specificities for different functional events. To overcome these limitations, we previously reported on an array-based approach to capture and quantitate antigen-specific T cells based on their adherence to pMHC complexes [16]. Here, we report a further development of this technology, in which we combined the high-throughput capture and activation of antigen-specific T cells described previously with the simultaneous analysis of the secretion of a wide variety of factors with single-cell resolution. Using this technique, we assess antigen-specific T cells from different vaccine recipients and analyze different functional profiles following antigen recognition in an attempt to explore the variability of clinical outcomes that is characteristic of tumor vaccine trials.

## Methods

### Peptides and Cell Lines

The peptides gp100 209–2M (IMDQVPFSV), MART1 M26 (ELAGIGILTV), tyrosinase 370D (YMDGTMSQV), gp100 209 (ITDQVPFSV), MART1 27–35 (AAGIGILTV), CMV pp65 495–503 (NLVPMVATV), EBV BMLF1 280–288 (GLCTLVAML), and influenza MP 58–66 (GILGFVFTL) were produced at the Protein and Nucleic Acid Facility at Stanford University (Stanford, California, United States).

CD8+ T cell clones were derived and maintained as previously described [17]. Briefly, clones were derived from melanoma patients or healthy donors expressing the human leukocyte antigen (HLA)-A2.1 MHC molecule. Clone 132.2 specifically binds gp100 209 or gp100 209–2M/HLA-A2.1. Clones 461.30 and 461.24 specifically bind MART 27–35 or M26/HLA-A2.1. Clone CMV94.3 specifically binds CMV pp65 495–503/HLA-A2.1 and was derived by fluorescence-activated cell sorter (FACS) separation of individual tetramer-positive cells from PBMCs from a healthy donor. T cell clones were cultured in CTL medium (Iscove's modified Dulbecco's medium, with 10% fetal calf serum, 2% human AB sera, and standard cell-culture concentrations of penicillin, streptomycin, and L-glutamine) supplemented with 50 U/ml IL-2. The clones were expanded by stimulation with phytohemagglutinin (Invitrogen, Carlsbad, California, United States) at a 1:100 dilution, followed by 14 d of culture in CTL medium with irradiated feeder cells and 50 U/ml of IL-2. Following expansion, clones were either cryopreserved or maintained in culture with CTL medium supplemented with 50 U/ml IL-2 or 2 ng/ml IL-15, and used within 2 wk. Cryopreserved cells were thawed at least 2 d prior to assays, and were suspended in CTL medium with 100 U/ml IL-2. At 1 d prior to experiments, the clones were transferred to fresh CTL medium without interleukins.

### Preparation of pMHC Class I

The pMHC tetramers were developed in the Davis lab and were prepared as previously described [11]. Alternatively, tetramers were purchased from Beckman Coulter (Allendale, New Jersey, United States). The pMHC dimers were purchased from BD Pharmingen (San Diego, California, United States) and prepared per manufacturer's protocol. All pMHC constructs were supplemented with glycerol prior to printing (2% final concentration).

### Vaccination Protocol

A randomized phase II trial for patients with resected stages IIC/III and IV melanoma who were HLA A*0201-positive and expressed at least one of the following was conducted: HMB-45 (gp100), tyrosinase, or Melan-A (MART-1). Informed consent was obtained from all patients. A total of 60 patients were randomly allocated to receive three peptides at 1 mg each (gp100 209–2M, tyrosinase 370D, and MART-1 M26) emulsified with the adjuvant Montanide ISA 51 in a 1:1 ratio by volume with: (A) IL-12 at 30 ng per kilogram body weight, (B) IL-12 at 100 ng per kilogram, and (C) IL-12 at 30 ng per kilogram with GM-CSF at 83 μg per peptide emulsified. All patients had a CT scan of the chest/abdomen/pelvis and brain MRI required to show no evidence of disease within 28 d of initiation of vaccine treatment. Injections were given at weeks 0, 2, 4, 6, 10, 14, 18, and 26, then week 50 for a total of nine injections over 1 y. Leukopheresis was performed just prior to the first injection and within 2 wk after injection number 8 (week 28) for immune response assays. Smaller blood samples were obtained at 3, 9, and 12 mo. Clinical specimens were frozen and stored prior to use, as previously described [18,19]. Tetramer flow cytometry on pretreatment blood samples showed no detectable melanoma antigen-specific T cells.

### Antibodies

Antibodies against human CD8 (HIT8a), HLA-A2 (BB7.2), IFNγ (NIB42, 4S.B3), TNFα (MAb1, MAb11), granzyme B (2CF/F5, GB11), GM-CSF (BVD2-23B6, BVD2-21C11), IL-2 (5344.111, B33–2), IL-4 (8D4–8, MP4-25D2), IL-5 (TRFK5, JES1-5A10), IL-10 (JES3-9D7, JES3-12G8), and IL-12p70 (20C2, C8.6) were purchased from BD Pharmingen (San Diego, California, United States); antibodies against IL-1a (4414.141, pAb BAF200), IL-1b (8516.311. pAb BAF201), IL-3 (4815.211, pAb BAF203), IL-6 (6708, pAb BAF206), IL-7 (7417, pAb BAF207), IL-13 (32116, pAb BAF213), IL-15 (34593, pAb BAF247), IL-17 (41809, pAb BAF317), lymphotactin (109001, pAb BAF695), IP-10/CXCL10 (33036.211, pAb BAF266), TGF-β1 (9005, 27240), TNFβ (5807, pAb BAF211), vascular endothelial growth factor (VEGF; pAb AF293NA, pAb BAF293), and VEGF-D (78902, 78923) were purchased from R&D Systems (Minneapolis, Minnesota, United States), and granzyme A (CLB-GA29, CLB-GA28) was purchased from Research Diagnostics Incorporated (Flanders, New Jersey, United States). Clone names are listed in parentheses, with biotinylated antibody clone names listed second, where appropriate. Polyclonal antibodies are noted with a pAb prefix.

### Preparation of pMHC Functional Microarrays

Libraries of pMHC/antibody mixtures were prepared as follows: Each of the pMHC constructs was mixed with a panel of antibodies against potentially secreted factors (Table 1), such that each mixture contained a single pMHC construct (2.5 mg/ml final concentration) and a single antibody against a secreted factor (0.5–1mg/ml final concentration). Each of these pMHC-based mixtures was supplemented with 2% glycerol. A second library with 0.5 mg/ml of either anti-human CD8 or anti-human HLA-A2 (instead of pMHC) and an antibody against a secreted factor was prepared as a nonactivating control. A volume of 12 μl of each mixture was loaded into a 384-well plate (MJResearch, Waltham, Massachusetts, United States) and arrayed in triplicate onto three-dimensional substrates composed of microscope slides coated with a polyacrylamide gel (Perkin Elmer, Boston, Massachusetts, United States), preprocessed according to the manufacturer's instructions. Samples were dispensed using a non-contact piezoelectric arrayer (Perkin Elmer), such that each spot contained ten drops of approximately 0.45 nl each. Printed proteins were immobilized within the gel substrate by incubating the slides for 48 h at 4 °C in a humid chamber. Following the immobilization, the arrays were placed in a dry slide box, sealed with tape, and stored at 4 °C until use. Arrays were tested for specific capture of secreted factors using defined concentrations of recombinant human factors (Quantikines, R&D Systems) incubated on an unused array for 30 min at room temperature, followed by 12 h at 4 °C. Arrays were then washed, developed, and imaged, as described below.

### Flow Cytometry Analysis

Patient PBMCs were analyzed for G209-2M-tetramer reactive cells by flow cytometry as described previously [13]. Briefly, cells were reacted with G209-2M-tetramer-PE (Beckman Coulter Immunomics Operations, San Diego, CA, United States) at 1:200 dilution for 20 min at room temperature, followed by anti-CD19 FITC (Caltag Laboratories, Burlingame, California, United States) and anti-CD8 PerCP-Cy5.5 (BD Biosciences, San Jose, California, United States) antibodies at final staining dilution of 1:40 and 1:20, respectively, for an additional 20 min. Cells were then washed and analyzed using a FACSCalibur flow cytometer (Beckton Dickinson, San Jose, California, United States). Approximately 10^5^ events were acquired from each sample and analyzed using FlowJo software (TreeStar, San Carlos, California, United States). Plotted CD19−, CD8+, tetramer+ lymphocytes were calculated as percent of total CD8+ lymphocytes for each sample.

### Binding and Secretion Assays

Binding and secretion assays were performed with either patient CD8+ T cells or cultured human CD8+ T cell clones. CD8+ T cells were isolated from 5 × 10^7^ PBMCs from patients on the above-described vaccine protocol, using a CD8− isolation column (Miltenyi Biotec). The CD8+ T cells were then resuspended in 200 μl of incubation medium (RPMI supplemented with 5% FCS, glutamine, and standard concentrations of penicillin and streptomycin). Alternatively, 1 × 10^6^ CD8+ T cell clones, as described above, were resuspended in 200 μl of incubation medium. For pMHC binding analysis, the single cell suspension was incubated on the pMHC array for 10–30 min at 20 °C. At the end of the incubation period, the array was washed in calcium- and magnesium-free PBS (CMF) to remove unbound cells and imaged as detailed below. To analyze cellular secretion, the cells were incubated on the array in 400 μl of incubation medium at 37 °C for 2 h (CD8+ T cell lines) or for 24 h (patient samples). To determine the secretion of factors, the arrays were washed in CMF and incubated in 200 μl of pooled biotinylated antibodies in staining medium (10% FCS in CMF) for 20 min at 20 °C. The biotinylated antibodies were each matched to a single, printed antibody specific against different epitopes of the same secreted factor. The final concentration of each biotinylated antibody was based upon concentrations recommended for ELISA or ELISpot, and titrated as necessary. After incubation with biotinylated antibodies, the array was washed twice in CMF and stained with 3.3 μg/ml streptavidin-phycoerythrin (BD Pharmingen) in 200 μl of staining medium for 20 min at 20 °C in the dark. The array was dip-washed twice again in CMF and then imaged as detailed below.

### Image Acquisition and Analysis

Imaging was performed using a Zeiss Axiovert-200 microscope (Oberkochen, Germany) fitted with a high-speed piezo electric z-motor stage (Applied Scientific Instrumentation, Eugene, Oregon, United States), a 10× Zeiss Fluor objective, a CCD camera (Roper Scientific, Trenton, New Jersey, United States), and dual excitation and emission filter wheels (Sutter Instruments, Novato, California, United States). DIC and Cy3 images were collected from each spot on the array. Image acquisition was controlled by Metamorph (Universal Imaging, Downingtown, Pennsylvania, United States). Image analysis, feature extraction, and data analysis were performed using Metamorph, ImageXpress (Molecular Devices, Union City, California, United States), and Matlab Software (The MathWorks, Natick, Massachusetts, United States).

### Analysis of Patient Data

Scoring of patient samples was performed in a blinded fashion. Coded samples were scored without prior information regarding patient age, sex, therapy, clinical, or immunological outcome. Scoring was based on a five-point scale (i.e., 0–4), with 0 representing background signal. A cell count score for IFNγ and TNFα secretion was based upon the number of responding cells per spot: 0, no response; 1, 1–5; 2, 6–10; 3, 11–20; and 4, more than 21 responding cells. A second score, for intensity, was based on average integrated pixel fluorescence over all replicate spots (after subtraction of the average integrated pixel fluorescence of control spots containing only pMHC). Each of the averaged intensities was normalized by a value greater than the highest intensity for that particular secreted factor, across all patients and expressed as a percentage of that value. The intensity score was assigned as follows: 0, 0%–5%; 1, 6%–25%; 2, 26%–50%; 3, 51%–76%; and 4, 77%–100%. A combined score for IFNγ and TNFα was obtained by adjusting the cell count score up or down by 1 if the intensity score was higher or lower than the cell count score. Scores for secreted factors lacking clear and consistent focal secretion across all patients (including granzyme B, IL-2, TGFβ, IL-1b, IL-6, GM-CSF, IL-1a, IL-3, IL-4, IL-5, IL-6, IL-7, IL-10, IL-12, IL-13, IL-15, IL-17, lymphotactin, IP-10, TNFβ, VEGF, VEGF-D, and granzyme A) received only intensity scores according to the following scale: 0, 0%–5%; 1, 6%–19%; 2, 20%–50%; 3, 51%–80%; and 4, 81%–100%. Six clinical specimens were initially tested using the pMHC functional array. Five additional specimens were tested to assess consistency of findings and expand the number of analyzed specimens.

## Results

### Specific Capture of Human Antigen-Specific T Cells Using a Readily Scalable pMHC Array

**Figure 1:**
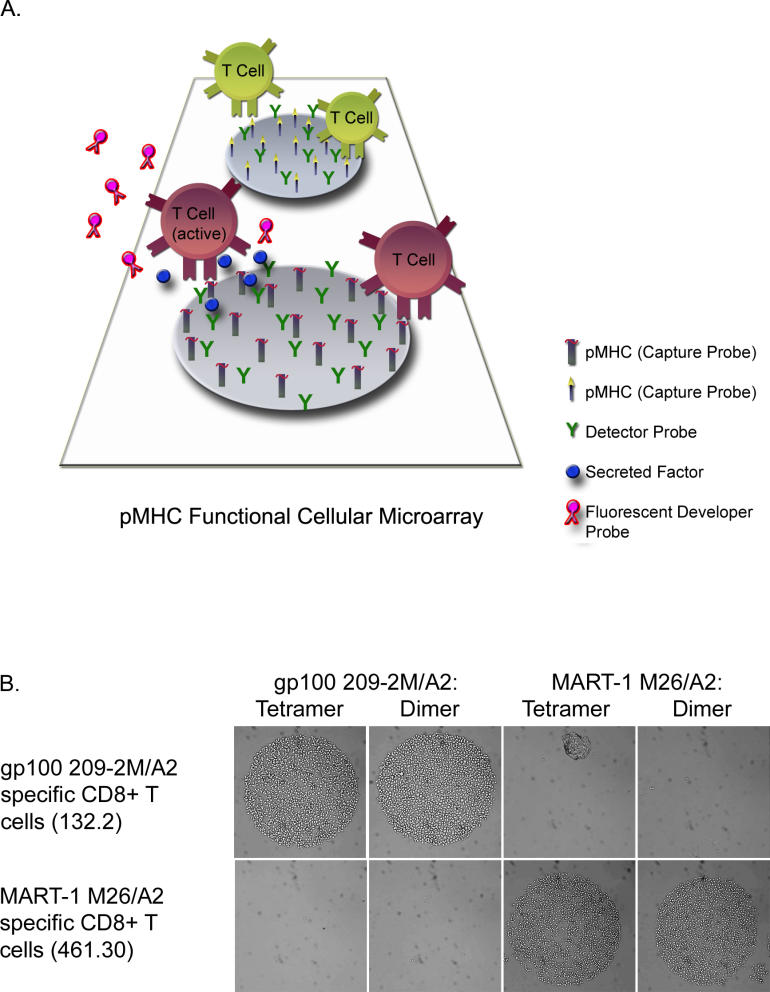
Peptide-MHC Cellular Microarrays (A) A functional pMHC microarray diagram illustrating the array format. An array of spots containing different capture probes (pMHC molecules) cospotted with detector probes, which are antibodies against potentially secreted factors. Antigen-specific T cells are captured by recognition of printed pMHC, and may become activated. Subsequent secretion of specific factors is captured by the printed detector probes. The presence of those factors is detected by labeled secondary antibodies (developer probes). (B) Specificity of pMHC T cell capture is peptide-specific. Human CD8+ lymphocyte clones 132.2 and 461.30 were incubated on duplicate microarrays, which included gp100-2M/HLA-A2.1 and MART-1 M26/HLA-A2.1 tetramer and dimer spots. 2M-specific 132.2 cells were exclusively captured by gp100 spots, and M26-specific 461.30 binding was restricted to MART-1 spots. Binding efficiency to pMHC tetramer or dimer of a given specificity was similar.

In our earlier work, we used pMHC tetramers spotted onto arrays to capture and quantitate specific T cells [16]. This has the disadvantage that the synthesis of pMHC tetramers is time consuming and not easily scalable to survey large numbers of different pMHC complexes. To address this problem, we used dimeric HLA-A2-immunoglobulin (Ig)-containing molecules (DimerX, BD Biosciences) [20] that lack peptide and are readily loaded with specific peptide antigens. In this way, dozens and potentially thousands of pMHCs can be made simultaneously. We tested the specificity of capture of these molecules using melanoma-specific antigens and two well-characterized human CD8+ T cell lines on pMHC microarrays printed on polyacrylamide-coated slides. The CD8+ T cell clones 132.2 and 461.30 were originally isolated from two patients vaccinated with gp100 209–2M peptide and MART1 M26 peptide [17] and expanded in vitro. Using flow cytometry, 132.2 stains exclusively with the gp100 209–2M/HLA-A2.1 tetramer while 461.30 stains with MART1 M26/HLA-A2.1, but not the gp100 209–2M/HLA-A2.1 tetramer. A microarray using pMHC constructs (gp100 209–2M/HLA-A2.1 tetramer, gp100 209–2M/HLA-A2.1 dimer-Ig, gp100 154/HLA-A2.1 dimer-Ig, MART1 M26/HLA-A2.1 tetramer, MART1 M26/HLA-A2.1 dimer-Ig and MART1 27/HLA-A2.1 dimer-Ig and monoclonal antibodies (anti-CD8a, and anti-HLA-A2) was constructed. For the assay, 132.2 (gp100-specific) and 461.30 (MART1-specific) cells were overlaid onto separate arrays. While both cells bound to the monoclonal antibody spots, 132.2 cells bound exclusively to the gp100 pMHC spots, while 461.30 cells bound only to the MART1 pMHC spots. Binding to specific tetramer and dimer spots was equivalent (Figure 1).

### pMHC Arrays Are Sensitive to Low-Frequency T Cell Populations

**Figure 2:**
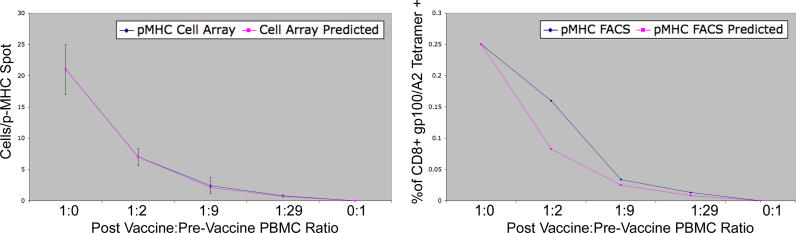
Sensitivity of pMHC-Specific T-Cell Detection The sensitivity of array-based T cell detection was compared to FACS-based detection. Patient PBMCs post-gp100 peptide vaccination were serially diluted in corresponding prevaccination sample. The dilution series was analyzed by both methods. Blue-labeled data correspond to the average number of bound cells per spot on the array (left graph) or the frequency of cells measured by FACS (right graph). The limit of detection in both methods was similar. Average number of bound cells per spot was obtained from five replicate spots on the same array. Error bars denote standard error of the mean across replicates. Red curves denote predicted results based on the values measured for the undiluted sample.

We compared the sensitivity of array-based detection to pMHC tetramer staining and flow cytometry for gp100 209–2M-specific CD8+ T lymphocytes from human clinical samples. PBMC samples collected from a patient pre- and post-gp100 peptide vaccination were used in this comparison. FACS analysis using tetramer staining indicated that the pre-vaccine sample was negative for gp100 209–2M, while the postvaccine sample contained 0.19% positive CD8+ T cells. To test the limits of detection in both methods, we diluted postvaccine CD8+ T cells in the gp100 209–2M negative prevaccine sample. CD8+ T cells were isolated from both samples using a depletion column and mixed at post-vaccine:pre-vaccine ratios of 1:0, 1:2, 1:9, 1:29, and 0:1. Each mixture was analyzed separately, using a pMHC microarray and tetramer/FACS. T cells captured on the microarray spots were counted and averaged over five identical gp100 209–2M/HLA-A2.1 spots. Both methods were able to detect antigen-specific T cells at fractional abundances as low as one cell in 10,000, or 0.01% of the CD8+ population (Figure 2). Cellular microarray binding variability was minimized by using the average number of cells bound over the five replicate spots printed on the same array. Results of the tetramer/FACS varied by the selection gate for forward scatter/side scatter, CD19+ dump and CD8+/tetramer+ staining. However, both cellular microarray and tetramer/FACS produced antigen-specific T cell frequencies that correlated well with serial dilution.

### Functional Profiling of Secreted T Cell Factors following Antigen Recognition

To study the functional responses of T cells after antigen recognition, we combined cell capture molecules (capture probes) with molecules that bind secreted factors (detector probes). Mixtures of a capture probe and detector probe were printed in triplicate on individual spots on the functional microarray. Seven different pMHC molecules (gp100 209–2M/HLA-A2.1, MART1 M26/HLA-A2.1, CMV pp65 495/HLA-A2.1, gp100 209/HLA-A2.1, influenza MP 58/HLA-A2.1, EBV BMLF1 280, and tyrosinase 370D) and four different monoclonal antibodies (anti-HLA-A2, anti-CD8, anti-CD3/anti-CD28) were used as capture probes. These were combined with detector probes composed of 26 different monoclonal antibodies specific for secreted factors (IL-1a, IL-1b, IL-2, IL-3, IL-4, IL-5, IL-6, IL-7, IL-10, IL-12, IL-13, IL-15, IL-17, IFNγ, TNFα, TNFβ, GM-CSF, granzyme B, granzyme A, TGFβ, VEGF, VEGF-D, lymphotactin, and IP10). pMHC functional microarrays capture antigen-specific T cells, provide an activating signal, and capture specific secreted factor as they are released by a given T cell. The secretion profile is visualized using a sandwich assay. A mixture of matched biotinylated monoclonal antibodies (developer probes) are applied to the array, followed by streptavidin-phycoerythrin. The identity of each secreted cytokine is determined from its location on the array. The specific capture and detection of soluble factors was confirmed by directly incubating matched, quantified soluble factors on the array.

**Figure 3:**
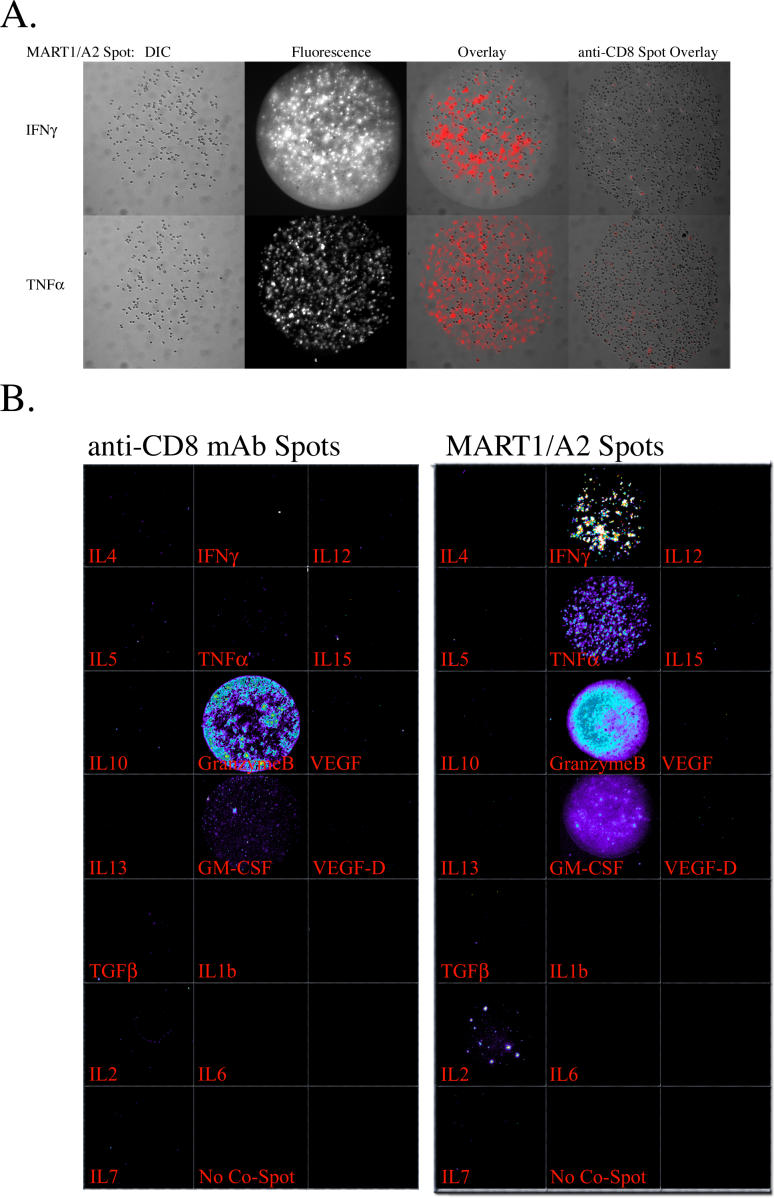
Profiling T Cell Function Clonally derived MART-1/A2 specific human CD8+ T cells were incubated on a functional pMHC microarray on which individual spots contained pMHC or a control anti-CD8 monoclonal antibody (i.e., capture probes) cospotted with a panel of antibodies against potentially secreted factors (i.e., detector probes). MART-1-specific cells were immobilized on both MART-1/HLA-A2.1 and anti-CD8 containing spots. Bound cells were further incubated at 37 °C for 2 h. Secreted factors were captured by the coprinted antibodies at close vicinity to the secreting cells and detected using matched, biotinylated antibodies. Some of the initially bound cells detached during the staining procedure. (A) Top and bottom rows display IFNγ and TNFα secretions, respectively, each detected at single-cell resolution. The fluorescence images (red) are overlaid onto the differential interference contrast light microscopy images in the rightmost two columns. Not all immobilized T cells secreted detectable factors. No T cell binding or fluorescence was detectable on irrelevant pMHC spots (unpublished data). (B) Secretion profile for 17 different factors. Capture probes are either anti-CD8 antibody (left) or MART1 M26/A2 (right). Cospotted detector probes are indicated for each spot. Secretion signal is shown in pseudocolor, representing fluorescence intensity. Secreted factor-specific scaling has been applied to maximize resolution.

Four CD8 T cell lines (132.2, 461.24, 461.30, and CMV94.3) were tested for their functional profiles following binding to their respective cognate antigen spot, or binding to a nonactivating spot (e.g. anti-CD8, anti-HLA-A2). Individually immobilized T cells could be visualized by light microscopy, and correlated with secreted factor captured from specific cells (Figure 3A). All antigen-specific T cell lines exhibited similar secretion profiles following antigen recognition, characterized by strong IFNγ, TNFα, granzyme A (unpublished data), and granzyme B secretion, and weaker GM-CSF and IL-2 secretion (Figure 3B). Baseline secretion of granzyme B and GM-CSF were detectable from cells bound to anti-CD8 and anti-HLA-A2 monoclonal antibody spots, but were much weaker than antigen-stimulated secretion.

### Detection of Heterogeneous Functional Profiles of Tumor-Associated Antigen-Specific T Cells Using a Functional pMHC Array

To gain insight into differences in patient responses to tumor vaccine therapy, we investigated the functional profiles of patient T cells on arrays of pMHC immobilized with different monoclonal antibodies directed against specific secreted factors. Eleven samples were taken from patients with resected stage IIC to IV malignant melanoma enrolled in a clinical trial involving gp100 209–2M, MART1 M26, and tyrosinase 370D peptide injection. These samples were taken by leukopheresis after eight injections of peptide and IL-12 adjuvant, 6 mo after the first injection (one sample was taken 12 mo after the first injection). CD8+ T cells were isolated from 5 × 10^7^ PBMCs using a negative isolation column and incubated on a functional pMHC array at 37 °C and 5% CO_2_ for 24 h. Secretion profiles were detected as described above.

**Figure 4:**
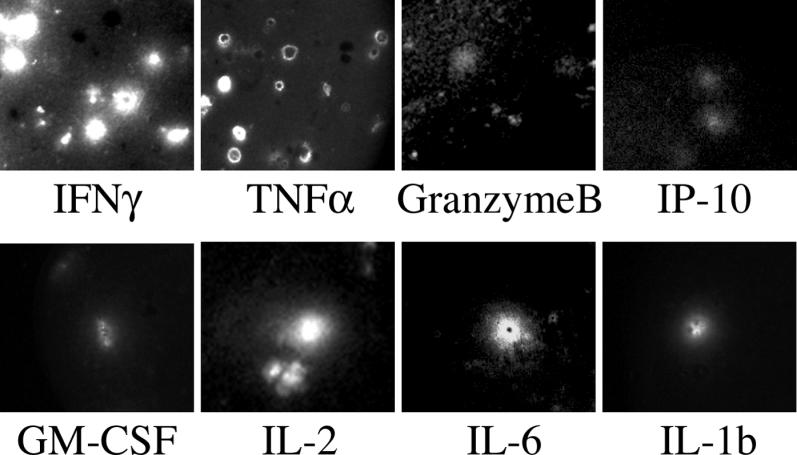
Anatomy of Cytokine Secretion Secreted cytokine captured as it is released from activated lymphocytes immobilized on a pMHC cellular microarray shows cytokine-specific configurations. Select representative patient samples are shown for each labeled cytokine to illustrate the patterns of secretion for each individual cytokine.

**Figure 5:**
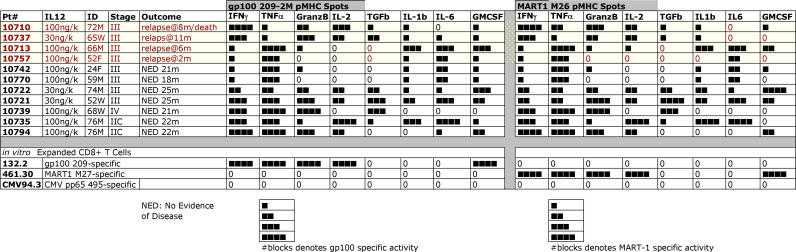
Heterogeneity of Melanoma-Associated Antigen-Specific T Cell Responses following Peptide Vaccination Eleven samples taken from patients enrolled in peptide vaccine trials were analyzed on pMHC functional microarrays. Patients received eight subcutaneous injections of peptides gp100 209–2M, MART1 M26, and tyrosinase 370D, along with adjuvant in a 6-mo period. Leukopheresis samples were collected after the eighth injection. Sample 10794 was collected from the same patient as 10735 after month 12. Functional profiles were generated by incubating patient CD8+ T lymphocytes on pMHC functional microarrays for 24 h at 37 °C and detecting the secreted factors with biotinylated secondary antibodies and streptavidin-phycoerythrin. Data were analyzed by automated fluorescence microscopy. Responses were scored on a five-point scale (0 to 4 bars), reflecting number of responders and overall fluorescent signal intensity per spot (Figure S1). Four bars indicate a strong response, and “0” indicates lack of a response. Each spot was printed in triplicate and analyzed individually. Patient clinical data are listed, including age and sex ( “ID”), stage of disease at enrollment (“Stage”), and outcome at follow-up (“Outcome”). Column labeled “IL12” specifies IL-12 adjuvant doses. Patient 10713 also received GM-CSF in addition to IL-12 adjuvant. Other secreted factors not shown include IL-4, IL-5, IL-10, IL-12p70, IL-1b, IL-3, IL-7, IL-13, IL-15, IL-17, lymphotactin, IP-10/CXCL10, TNFβ, VEGF, VEGF-D and granzyme A due to either lack of detectable secretion or limited analysis performed on only a fraction of the samples. In vitro restimulated cell lines directed against gp100 209 (132.2), MART1 M27 (461.30), or CMV pp65 495 (CMV94.3) were bound and secreted factors in response to gp100, MART1, and CMV (unpublished data), respectively.

**Figure 6:**
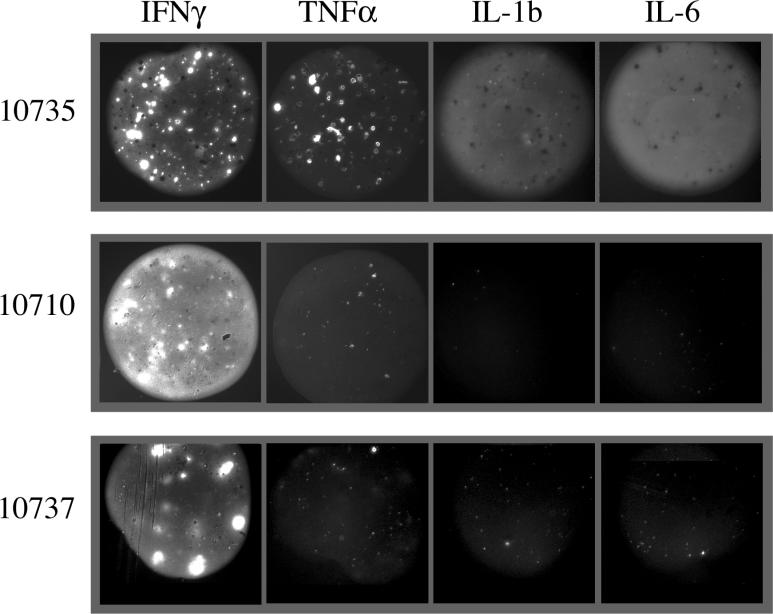
Differences in Functional Profiles between Three Patients with Different Clinical Outcomes gp100 209–2M spots co-spotted with anti-IFNγ, anti-TNFα, anti-IL1b, and anti-IL-6 are shown for three separate patient samples. Patient 10735, who remains free of disease at this writing, displays strong IFNγ, TNFα, IL-1b, and IL-6 secretion. Patients 10710 and 10737 show strong IFNγ secretion, but weak to no TNFα, IL-1b, and IL-6 secretion; both patients experienced disease recurrence soon after these samples were drawn. Note the diffuse pattern of IL-1b and IL-6 capture, which differs from the focal capture of IFNγ and TNFα.

**Figure 7:**
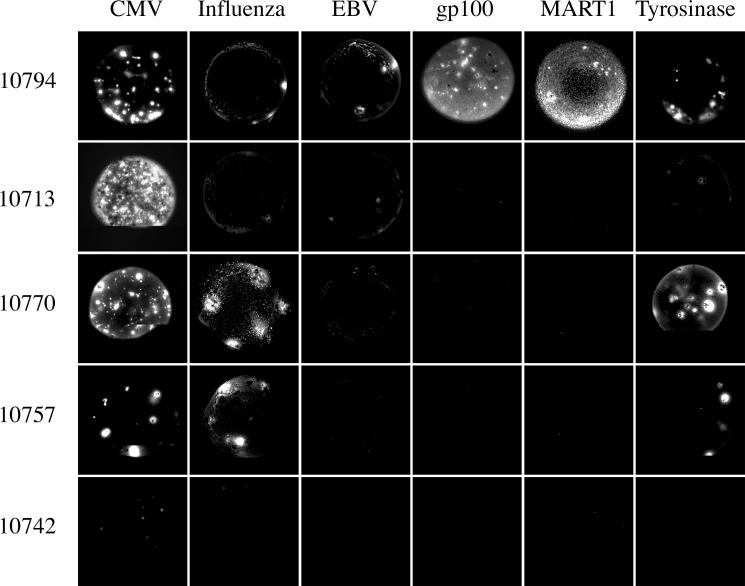
Antigen-Specific Profiles within Individual Patients CD8+ lymphocytes were isolated from PBMCs from patients 10794, 10713, 10770, 10757, and 10742, and incubated on a pMHC functional cellular microarray containing anti-IFNγ co-spotted with several different peptide-MHC (HLA-A2) complexes. These included melanoma-associated antigens gp100, MART-1, and tyrosinase, and viral antigens from cytomegalovirus, influenza virus, and Epstein-Barr virus (gp100 209–2M, MART1 M26, tyrosinase 370D, CMV pp65, influenza MP, and EBV BLF, respectively). The resulting IFNγ responses varied by antigen-specific T cell population.

Specific T cell immobilization was visible within 10 min, as described previously [16]. Cytokine secretion was detectable in each clinical sample as a phycoerythrin fluorescent signal on spots where specific cytokine capture antibodies had been printed. Secretion from individual immobilized cells resulted in the highly localized capture of cytokine. Different cytokines had different typical appearances on the array (Figure 4). IFNγ capture resembled a “starburst” pattern emanating from specifically bound cells, whereas TNFα capture resembled a thin ring around the cell. Some cytokines produced a diffuse signal, which may have resulted either from saturation of capture reagent on a given spot, or secretion from unbound bystander cells rather than specifically captured and activated cells responding to a printed antigen. We characterized the secretion profiles for each patient sample (Figure 5). Quantitative data extracted from each image included average spot intensity, reflecting the total amount of a given cytokine captured on a spot; spot intensity standard deviation, which reflects granularity of the developed signal; and the number of responding cells. The gp100-specific CD8+ T cells from four samples from three patients, 10721, 10739, 10735, and 10794 (10735 and 10794 were isolated from the same patient, at 6 and 12 mo, respectively) gave strong IFNγ and TNFα secretion signals. All three patients remain disease free 25, 21, and 22 mo, respectively, after initiating vaccine therapy. The gp100-specific CD8+ T cells from patient 10722 also responded with similar IFNγ and TNFα secretion. At this writing, this patient remains without evidence of disease progression after 25 mo. In contrast, four of six patients in whom gp100-specific CD8+ T cells mounted a strong IFNγ response, or a strong TNFα response, but not both (Figure 6), experienced a relapse of disease (patient samples 10710, 10737, 10713, and 10757 at 8, 11, 6, and 2 mo following initiation of vaccine therapy, respectively). Three out of four patients in this study that had strong GM-CSF secretion also remain free of disease at this writing. IL-1b and IL-6 were both strongly secreted by three patients, of which only one patient, 10713, has experienced recurrent disease. Patient response profiles to different antigens did not appear to be global. While some patients had detectable IFNγ responses to a plethora of different antigens (i.e., 10794), or had no detectable IFNγ response to the tested antigens (i.e., 10742), several patients had detectable responses to some antigens, but not others. Patients 10713, 10770, and 10757 failed to generate IFNγ secretion in response to gp100 or MART-1, but were capable of excellent IFNγ secretion in response to viral antigens or a different melanoma antigen, tyrosinase (Figure 7). Patient 10713 had no IFNγ response to gp100 209–2M/HLA-A2.1, MART1 M26/HLA-A2.1, or tyrosinase 370D/HLA-A2.1, but a very strong IFNγ response to a common CMV antigen, pp65 495/HLA-A2.1. These five samples were also tested for secretion of IFNγ, TNFα, granzyme B, IL-2, IL-1b, and IL-6 in response to gp100 209/HLA-A2.1 (wild-type peptide sequence). All five tested samples showed functional responses to wild-type gp100 209/HLA-A2.1 that mirrored the measured response to gp100 209–2M/HLA-A2.1 (unpublished data). Cytokine detector spots were calibrated with recombinant protein, or cells that secrete those factors. Despite immobilization of antigen-specific T cells, spots containing detectors against other secreted factors are not shown, due to lack of detectable signal.

## Discussion

Cellular immune responses are complex and multifaceted events involving a multitude of cell types, secreted factors, microenvironments, cell states, and temporal factors. Under certain conditions, such as cellular immune responses to viral infection, the response is capable of eradicating specific target cells that express aberrant proteins and programming a multicellular response by secreting proinflammatory cytokines. Both processes are mediated by factors secreted by T cells. However, endogenous or postvaccination immune responses to tumor-associated antigens are less predictable than the response to viral infections, and are generally less effective at eliminating the offending cells. This response may be due to lower-affinity TCRs expressed on tumor-associated antigen-specific T cells [17], inefficient T cell priming of these cells [21], and/or the presence of regulatory cytokines, factors, or cells [22]. This diversity is reflected in the wide range of clinical responses to experimental cancer vaccines [23]. Here, we provide a possible explanation for this heterogeneity. Using pMHC functional microarrays to analyze viable patient T cells, we demonstrate a wide variation in the responses of tumor-associated antigen-specific CD8+ T cells following tumor peptide vaccination.

The mechanism that controls which specific factors are secreted in response to T cell activation and the impact of different functional profiles on the overall clearance of tumor has not been established, but the remarkable heterogeneity of these responses highlights the importance and the challenge of understanding these relationships. The differences in T cell behavior, as measured by multiple secreted factors, may stem from differences between melanoma cells from different patients, and the regulation of their T cell responses to melanoma antigens. Melanoma cells themselves may shape the behavior of tumor-associated antigen-specific T cells via secreted factors, or cell-contact [24,25]. Recognizing differences in functional responses between patients and between different antigen-specific T cells within a patient should help guide the development of cancer vaccines by providing causal relationships between treatment and clinical outcome, thereby accelerating the testing of different vaccine strategies.

Although this study is limited in scope, and does not allow us to link specific secretion response profiles to clinical outcomes, the results do suggest hypotheses that can be tested in expanded studies. One such possibility is that active secretion of both IFNγ and TNFα in response to tumor-associated antigen recognition may be necessary for effective tumor clearance. As one of its many functions, TNFα can mediate inflammation and promote T cell priming [26–28]. Similarly, IFNγ can mediate increased MHC class I expression on the cell surface and increase CD4+ T cell help by shifting toward a T_H_1 phenotype [29,30]. Without the involvement of both factors, it is possible that a threshold level of inflammation and effector activity is not reached. Another possibility is that dual IFNγ and TNFα secretion are associated with other secreted factors of critical importance. What is most important at this point, however, is that the data described here show that cytotoxic cells with identical specificity can have diverse functional response profiles. This heterogeneity is likely to have profound consequences for the functional specificity and clinical efficacy of cellular immune responses and may mirror the heterogeneity in clinical outcomes seen in essentially all of the immunotherapy trials performed to date [31,32]. With the methodology described here, we should be in an excellent position to determine what immune response profile correlates best with a positive clinical outcome.

The functional responses seen here do not seem to fit into easily categorized “good” or “bad” response profiles. Each individual patient appears to have a unique signature of functional responses. This is in contrast to preliminary analysis of anti-influenza T cell responses following vaccination (DSC and MMD, unpublished data). Furthermore, the variation in the responses is both patient-specific and independently antigen-specific. For example, individuals who responded to gp100 209–2M with strong IFNγ, but weak TNFα secretion (e.g., patient 10710), could respond to MART1 M26 with very strong IFNγ and TNFα secretion, in the same CD8+ T cell sample analyzed on the same array. This was also true of the variation in CD8+ T cell responses to viral antigens, in the absence of vaccination. As all patients underwent a similar melanoma vaccination protocol, these findings suggest that T cell populations with different antigen specificities are differentially regulated in the same patient at the same time, perhaps a major source of the variation in functional responses. Interestingly, the strong IFNγ and TNFα response to gp100 209–2M/HLA-A2.1 and MART1 M26/HLA-A2.1 seen in patient sample 10735 (after 6 mo) was still present in sample 10794 (same patient, after month 12).

**Figure 8:**
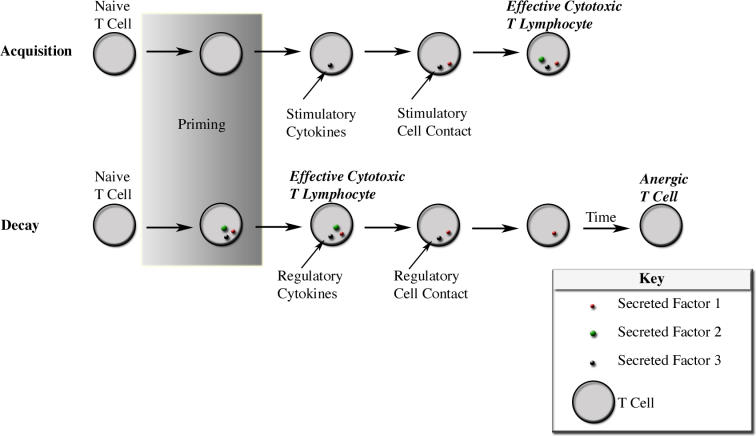
Two Models of T-Cell Function Acquisition and decay models depict two possible mechanisms that account for variability in the factors secreted by activated CD8+ T lymphocytes in response to antigen recognition. Acquisition refers to independent, sequential increases in responsiveness to activation, triggered by both cellular and secreted signals. Decay accounts for maximally functional T cells immediately upon completion of priming, after which signals, or time, lead to diminished responsiveness to activation.

The surprisingly multidimensional variation in the molecular specificity of the cellular immune response to a peptide vaccine raises the question of how and why these diverse variations arise. One possibility is that T cells acquire specific molecular programs based upon specific cues or signals accompanying or following the encounter with cognate antigen. Such signals could be mediated by secreted factors, or require cell-cell contact and might originate from antigen-presenting cells, CD4+ T cells, or a local complex inflammatory response. We refer to such a possible system as the “acquisition model,” in which incremental gains in their repertoire of functional responses (e.g., in the repertoire of effectors secreted in response to antigen stimulation) would change the CD8+ T cell's ability to effect killing and inflammation upon activation. An alternative possibility is that all T cells emerge from priming with the same level of functionality, capable of mediating a potent effector response. Following priming, their function could change with time, lack of stimulation, or the action of regulatory factors, causing them to slowly lose their ability to respond effectively to antigen recognition. We refer to this possibility as the “decay model” (Figure 8). Whether any stereotypical, ordered progression for either acquisition or decay of the response repertoire exists is unclear. However, our data do not support a single stereotyped progression, as T cells from some patients appear to respond with IFNγ, but not TNFα, while others respond with TNFα, but not IFNγ. One final possibility, an “intrinsic model,” is that the differences in functionality presented here are not evolving, but rather reflect the selection of T cells with specific predetermined molecular phenotypes. In this case, TCR affinity and other structural phenotypes might account for differences in function following activation. Changes in functional profiles in this model would reflect the emergence of new antigen-specific clones.

### Isolating Individual Events in Complex Immune System Responses

The cellular arm of the adaptive immune response is based on specific recognition of target antigens presented on the surface of altered cells and subsequent triggering of a complex scenario of responses, collectively termed effector function. To investigate the cellular immune response to antigen recognition, we have developed a high-throughput multiparametric platform that simultaneously captures antigen-specific T cells and facilitates parallel induction and monitoring of distinct secreted factors from multiple T cell specificities [33,34]. A similar approach that has been used for the capture and analysis of antigen-specific T cell clones was recently reported by Stone, et al. [35]. However, the technique described here differs from that of Stone and colleagues in several important aspects, including the selection of a surface with lower cellular binding characteristics and greater protein loading capacity. We have found these features to be critical to the detection of rare populations of antigen-specific T cells, and their secreted proteins, from clinical specimens. Detection of antigen-specific T cell populations on this platform compares favorably with approaches such as pMHC tetramer staining followed by flow cytometry. We have noted a similar level of sensitivity for reproducible detection of rare cell populations. Analysis of a single clinical sample on the pMHC cellular microarray includes isolation, quantitation, and activation of antigen-specific T cells, followed by characterization of secreted proteins with single-cell resolution. This type of analysis is impractical or impossible to perform with more traditional approaches, such as pMHC tetramer staining, ELISpot [36], and cytokine flow cytometry [37]. Unlike ELISpot assays or cytokine capture arrays, the pMHC microarray immobilizes specific cells prior to functional characterization. In addition, due to a higher concentration of the coprinted detector probe antibodies, the secreted factors are captured and subsequently detected in close proximity to the secreting cells, with minimal dilution. The resultant signal is detectable in a physical pattern that may provide further clues to their physiologic roles and mechanisms of action.

In some cases, the presence of unresponsive (i.e., nonsecreting) cells can also be determined based on a characteristic signature of a nonfluorescent, cell-shaped region embedded within a brighter region (see Figure 3). By combining isolation with activation, antigen-specific T cells can be studied under controlled environments, where the influence of a specific factor or cell type can be ascertained. Cospotting of additional membrane-bound ligands (e.g., B7–1, ICAM), or even secreted factors (e.g., IFNγ, TGFβ, IL-2, IL-15) may further help to elucidate the complex network of interactions underlying T cell reactivity or lack thereof. The spatial resolution of secreted factor detection on a pMHC microarray is sufficiently high to distinguish between different factors based on the characteristic signature of secretion. For example, the IFNγ signature appears as a focal secretion characterized by an intense core, beyond which the signal decays sharply (Figure 7). In contrast, the appearance of captured TNFα is characterized by a clearly demarcated ring that appears outside the edge of bound or previously bound cells. These patterns suggest that IFNγ secretion is polarized toward the target cell, whereas TNFα is not detectable at the contact interface; thus, it may be broadcasting a signal rather than engaging in a dialog with the target cell (as also indicated by work in murine T cells; M. Huse, personal communication). The spatial resolution of detected cytokine secretion also reveals marked differences in the quantity of cytokine secreted by different T cells of the same antigen specificity. However, it is unclear what mechanisms control the quantity of cytokine secreted by a given responding T cell, or the significance of higher levels of secretion. One may speculate that higher numbers of tumor-associated antigen-specific T cells that secrete larger quantities of effector cytokines favor a more effective antitumor response.

In humans, the T cell component of the immune system comprises a tremendous number and diversity of T cells with different antigen specificities. Profiling a large and diverse range of T cell specificities on a single pMHC array platform can allow more thorough interrogation and understanding of ongoing responses from a single clinical sample. Here, we tested a strategy for constructing very large pMHC arrays by using a hybrid MHC (class I):Ig dimer construct that can be easily loaded with an arbitrary HLA- restricted peptide. The success of this approach suggests that a printable library of diverse pMHC constructs can be prepared simply by loading many different peptides in parallel.

The ability to generate functional profiles of cells present in clinical samples is not limited to characterization of antigen-specific T cell responses. This type of approach can be applied using a wide range of cell adhesion and signaling molecules to specifically capture cells in heterogeneous populations, and profile the molecules they secrete in response to specific signals, with single-cell resolution. As is true with responding CD8+ T cells, all cells use a diverse vocabulary of secreted proteins to communicate with other cells and modify their environment. Thus, the profiles that microarrays of this kind can provide may give us insight into what different populations of cells are capable of communicating and how they can manipulate their environment. The patterns that emerge from this type of systematic analysis could provide us an understanding of the language of molecular communication among cells, and how groups of cells orchestrate their behavior at the tissue level. This immediate improvement in the resolution with which we can profile the functional characteristics of living human cells is likely to find clinical applications beyond tumor immunology.

## Supporting Information

Figure S1 Expanded gp100 and MART-1 Specific T Cell Functional Activity The data used to generate Figure 5 are shown in this figure.
(A) Number of cells responding per spot to gp100 or MART1 with secretion of IFNγ or TNFα.
(B) Average intensity value gp100 spots and MART1 spots for each individual secreted factor. Spot fluorescence intensity was measured from each spot and averaged over all replicates. Intensity values are then expressed as a percentage of an arbitrary value specific for each secreted factor.
(25 KB PDF).

**Patient Summary**

**Background**

Malignant melanoma is a common skin cancer that is frequently fatal. One type of treatment being tested is vaccination with peptides (very short lengths of proteins) that are found on the surface of melanoma cells, in an attempt to produce an immune response to the tumor, which will then clear the tumor. However, the success of this treatment has been quite varied; in particular it has been very hard to predict which patients will respond to treatments and which will not.

**Why Was This Study Done?**

The authors wanted to understand why some people respond to vaccination and others do not. One way of measuring the response to vaccination is to look at the T cells (part of the body's immune response) that are specific to the melanoma proteins and which are produced after vaccination, and to measure how active they are in various ways.

**What Did the Researchers Do and Find?**

The researchers developed a way of catching individual T cells from the blood of patients onto a surface, stimulating the cells, and then measuring how the cells responded by measuring how much of various substances the cells produced. They tested the responses of T cells from ten patients who had been enrolled in a trial of vaccination against melanoma and found a wide variation in how much of various substances the patients' cells secreted; in patients whose tumors did not get bigger, it seemed necessary for the cells to secrete two particular compounds—interferon gamma and tumor necrosis factor alpha.

**What Do These Findings Mean?**

Unlike responses to vaccination for infectious diseases, the response to tumor vaccination is highly variable between patients. Studying the basis of these different responses may guide the future development of more effective vaccines.

**Where Can I Get More Information Online?**

Medline plus has links to many pages of information on melanoma:

http://www.nlm.nih.gov/medlineplus/melanoma.html

Cancer Bacup in the UK also has information for patients:

http://www.cancerbacup.org.uk/Cancertype/Melanoma/Resourcessupport/PatientInformationGuide

The National Cancer Institute has a page containing links to information on melanoma:

http://www.cancer.gov/cancertopics/types/melanoma

The National Institutes of Health has a searchable index of ongoing clinical trials for melanoma:

http://www.clinicaltrials.gov/ct/screen/SimpleSearch

## Abbreviations

CMF: calcium- and magnesium-free phosphate-buffered saline
DIC: differential interference contrast light microscopy
FACS: fluorescence-activated cell sorter
FCS: fetal calf serum
GM-CSF: granulocyte-macrophage colony-stimulating factor
HLA: human leukocyte antigen
IFN: interferon
Ig: immunoglobulin
MHC: major histocompatibility complex
NED: no evidence of disease
PBMC: peripheral blood mononuclear cell
pMHC: peptide-MHC
TCR: T cell receptor
TNF: tumor necrosis factor
VEGF: vascular endothelial growth factor

## References

[pmed-0020265-b01] Rosenberg SA (2001). Progress in human tumour immunology and immunotherapy. Nature.

[pmed-0020265-b02] van der Bruggen P, Traversari C, Chomez P, Lurquin C, De Plaen E (1991). A gene encoding an antigen recognized by cytolytic T lymphocytes on a human melanoma. Science.

[pmed-0020265-b03] Lee PP, Yee C, Savage PA, Fong L, Brockstedt D (1999). Characterization of circulating T cells specific for tumor-associated antigens in melanoma patients. Nat Med.

[pmed-0020265-b04] Talebi T, Weber JS (2003). Peptide vaccine trials for melanoma: Preclinical background and clinical results. Semin Cancer Biol.

[pmed-0020265-b05] Kessels HW, Wolkers MC, Schumacher TN (2002). Adoptive transfer of T-cell immunity. Trends Immunol.

[pmed-0020265-b06] Andersen MH, Gehl J, Reker S, Pedersen LO, Becker JC (2003). Dynamic changes of specific T cell responses to melanoma correlate with IL-2 administration. Semin Cancer Biol.

[pmed-0020265-b07] Nasi ML, Lieberman P, Busam KJ, Prieto V, Panageas KS (1999). Intradermal injection of granulocyte-macrophage colony-stimulating factor (GM-CSF) in patients with metastatic melanoma recruits dendritic cells. Cytokines Cell Mol Ther.

[pmed-0020265-b08] Kirkwood JM, Manola J, Ibrahim J, Sondak V, Ernstoff MS (2004). A pooled analysis of eastern cooperative oncology group and intergroup trials of adjuvant high-dose interferon for melanoma. Clin Cancer Res.

[pmed-0020265-b09] Phan GQ, Yang JC, Sherry RM, Hwu P, Topalian SL (2003). Cancer regression and autoimmunity induced by cytotoxic T lymphocyte-associated antigen 4 blockade in patients with metastatic melanoma. Proc Natl Acad Sci U S A.

[pmed-0020265-b10] Pardoll D, Allison J (2004). Translation of cancer immunotherapies. Nat Med.

[pmed-0020265-b11] Altman JD, Moss PA, Goulder PJ, Barouch DH, McHeyzer-Williams MG (1996). Phenotypic analysis of antigen-specific T lymphocytes. Science.

[pmed-0020265-b12] Maecker HT, Dunn HS, Suni MA, Khatamzas E, Pitcher CJ (2001). Use of overlapping peptide mixtures as antigens for cytokine flow cytometry. J Immunol Methods.

[pmed-0020265-b13] Rubio V, Stuge TB, Singh N, Betts MR, Weber JS (2003). Ex vivo identification, isolation and analysis of tumor-cytolytic T cells. Nat Med.

[pmed-0020265-b14] Speiser DE, Pittet MJ, Guillaume P, Lubenow N, Hoffman E (2004). Ex vivo analysis of human antigen-specific CD8+ T-cell responses: Quality assessment of fluorescent HLA-A2 multimer and interferon-gamma ELISPOT assays for patient immune monitoring. J Immunother.

[pmed-0020265-b15] Johnson TR, Massey RJ, Deinhardt F (1972). Lymphocyte and antibody cytotoxicity to tumor cells measured by a micro-51 chromium release assay. Immunol Commun.

[pmed-0020265-b16] Soen Y, Chen DS, Kraft DL, Davis MM, Brown PO (2003). Detection and characterization of cellular immune responses using peptide-MHC microarrays. PLoS Biol.

[pmed-0020265-b17] Stuge TB, Holmes SP, Saharan S, Tuettenberg A, Roederer M (2004). Diversity and recognition efficiency of T cell responses to cancer. Plos Med.

[pmed-0020265-b18] Wong R, Lau R, Chang J, Kuus-Reichel T, Brichard V (2004). Immune responses to a class II helper peptide epitope in patients with stage III/IV resected melanoma. Clin Cancer Res.

[pmed-0020265-b19] Lee P, Wang F, Kuniyoshi J, Rubio V, Stuges T (2001). Effects of interleukin-12 on the immune response to a multipeptide vaccine for resected metastatic melanoma. J Clin Oncol.

[pmed-0020265-b20] Dal Porto J, Johansen TE, Catipovic B, Parfiit DJ, Tuveson D (1993). A soluble divalent class I major histocompatibility complex molecule inhibits alloreactive T cells at nanomolar concentrations. Proc Natl Acad Sci U S A.

[pmed-0020265-b21] Zimmermann VS, Bondanza A, Monno A, Rovere-Querini P, Corti A (2004). TNF-alpha coupled to membrane of apoptotic cells favors the cross-priming to melanoma antigens. J Immunol.

[pmed-0020265-b22] Viguier M, Lemaitre F, Verola O, Cho MS, Gorochov G (2004). Foxp3 expressing CD4+CD25(high) regulatory T cells are overrepresented in human metastatic melanoma lymph nodes and inhibit the function of infiltrating T cells. J Immunol.

[pmed-0020265-b23] Brichard VG, Gerard C (2003). Melanoma vaccines: Achievements and perspectives. Forum (Genova).

[pmed-0020265-b24] Zha YY, Blank C, Gajewski TF (2004). Negative regulation of T-cell function by PD-1. Crit Rev Immunol.

[pmed-0020265-b25] Dummer W, Bastian BC, Ernst N, Schanzle C, Schwaaf A (1996). Interleukin-10 production in malignant melanoma: Preferential detection of IL-10-secreting tumor cells in metastatic lesions. Int J Cancer.

[pmed-0020265-b26] Kwon B, Kim BS, Cho HR, Park JE, Kwon BS (2003). Involvement of tumor necrosis factor receptor superfamily(TNFRSF) members in the pathogenesis of inflammatory diseases. Exp Mol Med.

[pmed-0020265-b27] Prevost-Blondel A, Roth E, Rosenthal FM, Pircher H (2000). Crucial role of TNF-alpha in CD8 T cell-mediated elimination of 3LL-A9 Lewis lung carcinoma cells in vivo. J Immunol.

[pmed-0020265-b28] Beutler BA (1999). The role of tumor necrosis factor in health and disease. J Rheumatol Suppl.

[pmed-0020265-b29] Groettrup M, Khan S, Schwarz K, Schmidtke G (2001). Interferon-gamma inducible exchanges of 20S proteasome active site subunits: Why?. Biochimie.

[pmed-0020265-b30] Bernabei P, Coccia EM, Rigamonti L, Bosticardo M, Forni G (2001). Interferon-gamma receptor 2 expression as the deciding factor in human T, B, and myeloid cell proliferation or death. J Leukoc Biol.

[pmed-0020265-b31] Chen DS, Davis MM (2005). Cellular immunotherapy: Antigen recognition is just the beginning. Springer Semin Immunopathol.

[pmed-0020265-b32] Antonia S, Mule JJ, Weber JS (2004). Current developments of immunotherapy in the clinic. Curr Opin Immunol.

[pmed-0020265-b33] Chen DS, Soen Y, Davis MM, Brown PO (2004). Functional and molecular profiling of heterogeneous tumor samples using a novel cellular microarray. J Clin Oncol.

[pmed-0020265-b34] Soen Y, Chen DS, Stuge TB, Weber JS, Lee PP (2004). A novel cellular microarray identifies functional deficiences in tumor-specific T cell responses. J Clin Oncol.

[pmed-0020265-b35] Stone JD, Demkowicz WE, Stern LJ (2005). HLA-restricted epitope identification and detection of functional T cell responses by using MHC-peptide and costimulatory microarrays. Proc Natl Acad Sci U S A.

[pmed-0020265-b36] Miyahira Y, Murata K, Rodriguez D, Rodriguez JR, Esteban M (1995). Quantification of antigen specific CD8+ T cells using an ELISPOT assay. J Immunol Methods.

[pmed-0020265-b37] Murali-Krishna K, Altman JD, Suresh M, Sourdive D, Zajac A (1998). In vivo dynamics of anti-viral CD8 T cell responses to different epitopes. An evaluation of bystander activation in primary and secondary responses to viral infection. Adv Exp Med Biol.

[pmed-0020265-b38] Trapani JA, Sutton VR (2003). Granzyme B: Pro-apoptotic, antiviral and antitumor functions. Curr Opin Immunol.

[pmed-0020265-b39] Lieberman J, Fan Z (2003). Nuclear war: The granzyme A-bomb. Curr Opin Immunol.

[pmed-0020265-b40] Catalfamo M, Henkart PA (2003). Perforin and the granule exocytosis cytotoxicity pathway. Curr Opin Immunol.

[pmed-0020265-b41] Schroder K, Hertzog PJ, Ravasi T, Hume DA (2004). Interferon-gamma: An overview of signals, mechanisms and functions. J Leukoc Biol.

[pmed-0020265-b42] Aggarwal BB (2003). Signalling pathways of the TNF superfamily: A double-edged sword. Nat Rev Immunol.

[pmed-0020265-b43] Lu L, Zhu J, Zheng Z, Yan M, Xu W (1998). Jak-STAT pathway is involved in the induction of TNF-beta gene during stimulation by IL-2. Eur J Immunol.

[pmed-0020265-b44] Kaijzel EL, Bayley JP, van Krugten MV, Smith L, van de Linde P (2001). Allele-specific quantification of tumor necrosis factor alpha (TNF) transcription and the role of promoter polymorphisms in rheumatoid arthritis patients and healthy individuals. Genes Immun.

[pmed-0020265-b45] Rosenwasser LJ (1998). Biologic activities of IL-1 and its role in human disease. J Allergy Clin Immunol.

[pmed-0020265-b46] Gronvik KO, Andersson J (1980). The role of T cell growth stimulating factors in T cell triggering. Immunol Rev.

[pmed-0020265-b47] Benczik M, Gaffen SL (2004). The interleukin (IL)-2 family cytokines: Survival and proliferation signaling pathways in T lymphocytes. Immunol Invest.

[pmed-0020265-b48] Heinrich PC, Behrmann I, Haan S, Hermanns HM, Muller-Newen G (2003). Principles of interleukin (IL)-6-type cytokine signalling and its regulation. Biochem J.

[pmed-0020265-b49] Novelli F, Casanova JL (2004). The role of IL-12, IL-23 and IFN-gamma in immunity to viruses. Cytokine Growth Factor Rev.

[pmed-0020265-b50] Weng NP, Liu K, Catalfamo M, Li Y, Henkart PA (2002). IL-15 is a growth factor and an activator of CD8 memory T cells. Ann N Y Acad Sci.

[pmed-0020265-b51] Akira S (2000). The role of IL-18 in innate immunity. Curr Opin Immunol.

[pmed-0020265-b52] Hamilton JA (2002). GM-CSF in inflammation and autoimmunity. Trends Immunol.

[pmed-0020265-b53] Widmer MB, Morrissey PJ, Goodwin RG, Grabstein KH, Park LS (1990). Lymphopoiesis and IL-7. Int J Cell Cloning.

[pmed-0020265-b54] Farber JM (1997). Mig and IP-10: CXC chemokines that target lymphocytes. J Leukoc Biol.

[pmed-0020265-b55] Lavender P, Cousins D, Lee T (2000). Regulation of Th2 cytokine gene transcription. Chem Immunol.

[pmed-0020265-b56] Mocellin S, Marincola F, Rossi CR, Nitti D, Lise M (2004). The multifaceted relationship between IL-10 and adaptive immunity: Putting together the pieces of a puzzle. Cytokine Growth Factor Rev.

[pmed-0020265-b57] Wahl SM, Chen W (2003). TGF-beta: How tolerant can it be?. Immunol Res.

[pmed-0020265-b58] Mukaida N, Harada A, Matsushima K (1998). Interleukin-8 (IL-8) and monocyte chemotactic and activating factor (MCAF/MCP-1), chemokines essentially involved in inflammatory and immune reactions. Cytokine Growth Factor Rev.

[pmed-0020265-b59] Nishi J, Maruyama I (2000). Increased expression of vascular endothelial growth factor (VEGF) in Castleman's disease: Proposed pathomechanism of vascular proliferation in the affected lymph node. Leuk Lymphoma.

[pmed-0020265-b60] Li X, Eriksson U (2001). Novel VEGF family members: VEGF-B, VEGF-C and VEGF-D. Int J Biochem Cell Biol.

