# Supplementary material for: Marked Differences in Human Melanoma Antigen-Specific T Cell Responsiveness after Vaccination Using a Functional Microarray
Source: PLoS Med. 2005 Sep 20;2(10):e265. doi: 10.1371/journal.pmed.0020265 (PMC1216330; doi:10.1371/journal.pmed.0020265)
Supplement: Supplementary file 1 [file pmed.0020265.sg001.pdf]

A.

# of cellular responders/spot

	gp100 209-2M pMHC Cellul		MART1 M26 pMHC Cellular Microarray	
**Pt#**	**IFNγ**	**TNFα**	**IFNγ**	**TNFα**
**10710**	22	5	19	13
**10737**	21	4	24	2
**10713**	1	22	1	30
**10757**	2	11	1	7
**10742**	3	11	4	15
**10770**	3	14	5	19
**10739**	28	34	34	39
**10721**	21	26	11	11
**10722**	11	11	12	12
**10735**	51	63	31	52
**10794**	34	57	21	33

B.

intensity value percentile/spot

	gp100 209-2M pMHC Cellular Microarray							
**Pt#**	**IFNγ**	**TNFα**	**GranzB**	**IL-2**	**TGFb**	**IL-1b**	**IL-6**	**GM-CSF**
**10710**	75	20	49	51	11	6	4	6
**10737**	15	6	62	20	20	12	8	8
**10713**	18	99	11	5	0	68	68	54
**10757**	16	75	2	2	0	6	30	10
**10742**	16	75	8	4	0	7	33	12
**10770**	17	74	4	4	0	8	24	6
**10739**	15	85	99	1	65	0	0	2
**10721**	32	17	62	34	77	36	57	20
**10722**	6	10	26	22	29	15	10	23
**10735**	56	55	9	84	11	71	78	6
**10794**	99	98	70	34	1	1	7	40

	MART1 M26 pMHC Cellular Microarray							
**Pt#**	**IFNγ**	**TNFα**	**GranzB**	**IL-2**	**TGFb**	**IL-1b**	**IL-6**	**GM-CSF**
**10710**	95	41	76	48	11	12	0	8
**10737**	6	3	30	23	13	10	4	5
**10713**	17	96	13	6	5	67	71	47
**10757**	16	75	0	2	0	3	21	4
**10742**	16	76	11	3	1	7	29	9
**10770**	17	76	5	2	0	11	28	1
**10739**	15	88	99	2	64	0	0	2
**10721**	22	25	89	28	89	49	77	33
**10722**	7	27	43	20	28	24	19	99
**10735**	36	72	13	86	12	87	88	5
**10794**	74	90	59	28	2	3	5	41

Supplemental Figure 1.
